# Adjunctive Effect of Green Tea Mouthwash Prepared at Different Steeping Temperatures on Gingivitis: A Triple-Blind Randomized Controlled Clinical Trial

**DOI:** 10.3390/dj9120139

**Published:** 2021-11-30

**Authors:** Hani T. Fadel, Alhanouf M. AlRehaili, Mona M. AlShanqiti, Afnan A. Alraddadi, Alhanouf M. Albolowi

**Affiliations:** 1 Division of Periodontology, Department of Preventive Dental Sciences, College of Dentistry, Taibah University, Al Madinah Al Munawwarah 41511, Saudi Arabia; 2 Ministry of Health, Bisha 67841, Saudi Arabia; hofe200@gmail.com; 3 Private Practice, Al Madinah Al Munawwarah 42243, Saudi Arabia; alshanqiti.mona@gmail.com (M.M.A.); alhanouf.mousa@gmail.com (A.M.A.); 4 Ministry of Health, Sakaka 72345, Saudi Arabia; afnaan.a95@gmail.com

**Keywords:** dental plaque, gingivitis, green tea, mouthwash, temperature

## Abstract

Purpose: To compare the effect of green tea mouthwashes prepared at different steeping temperatures as adjuncts to mechanical plaque control on gingivitis. Methods: Forty-five women with gingivitis participated in this 4-week randomized controlled clinical trial. They received professional mechanical plaque control and rinsed daily with either warm green tea, hot–cold green tea or placebo. Dental plaque control record (PCR) and gingival bleeding indices (GBI) were recorded at baseline and 7, 14 and 28 days after. Results: Participants’ mean age was 20.7 ± 2 years. The mean scores for the PCR and GBI at baseline were 82.4 ± 19 and 85.8 ± 7, respectively. All groups showed significant reduction in PCR and GBI between Days 0 and 28 (*p* < 0.01). No significant differences in PCR were observed between the groups at any of the examinations (*p* > 0.01). The warm green tea group demonstrated significantly lower GBI at all examinations compared to the hot–cold group (*p* < 0.01). Conclusions: Within study limits, green tea-made mouthwashes significantly reduced plaque and gingivitis when used as adjuncts to mechanical plaque control. The green tea mouthwash prepared in warm water demonstrated significantly higher efficacy in lowering gingivitis compared to that prepared in hot water followed by ice.

## 1. Introduction

Since the dawn of humanity and before the development of the pharmaceutical industry, herbal products were used for preventing and treating various conditions and common diseases [1]. They demonstrated wide biological activities, high safety margin, and low costs contrary to chemical drugs. Furthermore, conventional drugs are known to cause numerous side effects, and continuous intake of antibiotic drugs has resulted in antibiotic resistance [1]. Throughout history, a number of herbal medicines have been popularly used as dietary supplements such as mint, basil, parsley and ginger at the time of the ancient Greeks, and, infamously, green tea during the ancient Chinese era (≈2700 BC) [1].

Originally, the term “Tea” refers to the shrub *Camellia sinensis*. It is amongst the most consumed beverages in the world alongside water, coffee, and carbonated soft drinks [2]. Green tea has been used either as a beverage, a mouthwash, a local drug delivery device or as a chewing gum [2]. Scientific evidence indicates that green tea is indeed beneficial to health, and that many of the components of tea have specific health-promoting effects [2]. A number of epidemiological surveys demonstrated that green tea is linked to a lower incidence of several pathological conditions, including oral conditions, cardiovascular disease, stroke, obesity and cancer [3].

Maintenance of oral health contributes to the overall quality of life of an individual [4,5]. The two most common oral diseases, i.e., dental caries and periodontal diseases, are mostly plaque induced and are essentially preventable in nature [6,7]. Mechanical and chemical plaque control methods have proven to be successful in treating and preventing gingivitis [8], which if not managed in due course may progress to periodontitis [7].

Interestingly, green tea has been used as an adjunct in the treatment of plaque-induced gingivitis and showed to have desirable effects on clinical and biological parameters, providing a potentially natural and affordable alternative in the process [9]. A number of clinical studies showed that applying green tea as a chewing gum or mouthwash improved plaque and bleeding scores, reduced salivary IL-1β levels and reduced volatile sulfur compound (VSC) levels effectively compared to placebo [10,11]. In fact, chlorhexidine and green tea mouthwashes were found to be equally effective in reducing plaque and gingival inflammation [12]. However, available reports are still lacking specific details regarding preparation of such custom-made natural remedies [13].

Traditionally, green tea is prepared by brewing the leaves in water, i.e., steeping, at a temperature between 70 and 100 °C. Other steeping temperatures have also been reported. Differences in beverage temperatures had variable manifestations on erosive side effects and cariogenic microbial counts, which could be attributed to consequent changes in certain properties [14,15]. However, no studies to date have compared the adjunctive effect of green tea mouthwashes, prepared at different steeping temperatures, in managing plaque-induced gingivitis. The objective of this study was thus to compare the effect of green tea mouthwashes prepared at different steeping temperatures as adjuncts to mechanical plaque control in individuals with plaque-induced gingivitis. The null hypothesis was that differences in steeping temperature do not significantly influence the adjunctive effect of green tea mouthwash on gingivitis.

## 2. Materials and Methods

### 2.1. Study Design and Sample

This was a triple-blind, 4-week, prospective, randomized controlled clinical trial, which utilized parallel interventions in three groups that were equally randomized in an initial 1:1:1 ratio. It was approved by the Taibah University College of Dentistry Research Ethics Committee (TUCD-RC) (Registration No. 20181112, Date: 27 November 2018). It abides by the ethical guidelines for research involving human participants according to the Declaration of Helsinki [16], and follows the CONSORT guidelines for reporting randomized controlled clinical trials [17]. The study was also registered on ClinicalTrials.gov (Identifier: NCT04484792). The study was performed between November 2018 and December 2019.

**Figure 1:**
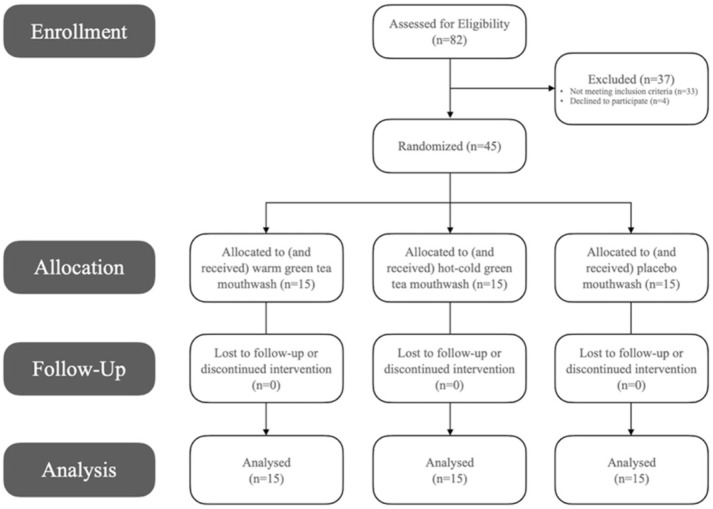
Flow diagram demonstrating enrollment, exclusion, group allocation and follow-up of study participants.

Out of 82 initially assessed, the study included 45 eligible female recruits between the ages of 18 and 35 years, with a clinical diagnosis of dental plaque-induced gingivitis on an intact periodontium, non-mediated by systemic or local factors according to the 2017 World Workshop on the Classification of Periodontal and Peri-Implant Diseases and Conditions, i.e., no signs of radiographic alveolar bone loss, probing pocket depths ≤3 mm, bleeding on probing ≥10% [18]. Particularly, those with bleeding on probing >70% were recruited to include more severe cases [18] (Figure 1). Periodontitis or non-periodontitis patients with a reduced periodontium, those with systemic conditions, pregnant ladies or breast-feeders, smokers, those under regular medication or who have received antibiotics during the past month, those who underwent any form of periodontal therapy within the past 6 months, or individuals regularly using mouthwash or any other chemical plaque control agent aside from their daily normal toothpaste, were all excluded from the study (Figure 1). Participants were patients attending the clinics at the Taibah University Dental College and Hospital (TUDCH), AlMadinah AlMunawwarah, Saudi Arabia. AlMadinah is the second holy city in Saudi Arabia, with a diverse growing population of over 1.1 million. Taibah University is one of two main governmental universities in the region, comprising 28 different colleges over three campuses. Moreover, TUDCH is recognized as a tertiary care dental center, accommodating approximately 8000 new patients each year, across various dental specialties. A dental office chair with an optimal light source were used for clinical examinations in this study.

### 2.2. Solution Preparation

The warm green tea mouthwash in Group A was prepared by soaking a green tea bag (Rabea Green Tea Pure Natural, Baeshen & Co., Jeddah, Saudi Arabia) in 100 mL of warm water at 40 °C for 5 min [19], while the hot–cold green tea mouthwash in Group B was prepared by soaking the green tea bag in 100 mL of boiled water for 5 min, followed by addition of three ice cubes (5 × 3 × 3 cm) [20]. The placebo mouthwash in Group C was regular bottled drinking water.

### 2.3. Study Outline

At baseline (Day 0), all 45 participants had an oral clinical examination involving the registration of selected oral health clinical parameters from all teeth, namely the gingival bleeding index (GBI) [21] and the plaque control record (PCR) [22], i.e., the primary study outcomes, which were chosen for simplicity and applicability in clinical settings and that accurately reflect periodontal health [23]. These were recorded using a periodontal probe and a disclosing agent, performed by a single assessor following training and calibration by an experienced periodontist (HTF). All participants were then subjected to a one-time supra-gingival scaling and tooth polishing session by a treating dentist, and received oral hygiene instructions including the use of the toothbrush, standard toothpaste (Signal Cavity Fighter, Unilever, Jeddah, Saudi Arabia), interdental cleaning using dental floss and rinsing with 7 mL of the provided mouthwash twice a day for 1 min. Participants were randomly allocated via block randomization utilizing a random sequence generator (Randomness and Integrity Services Ltd., Dublin, Ireland) into one of three groups with 15 participants in each: the warm green tea mouthwash group (A), the hot–cold green tea mouthwash group (B), and the placebo group (C). The participants, the assessor and the treating dentist were all blinded with regards to participants’ group allocation, while the examining/treating dentist and the registering nurse were blinded after the allocation. Scaling and polishing were only performed at the first visit (Day 0), while registration of oral health clinical parameters, and reinforcement of oral hygiene instructions were repeated on Days 7, 14 and 28 [24].

### 2.4. Data Analysis

Descriptive statistics in the form of medians, means and standard deviations were used to present quantitative data. Similarly, qualitative data were presented as frequency distributions and percentages. Inferential statistics were performed after completion of the 4-week trial period. Particularly, One-Way ANOVA was used to compare mean PCR and GBI scores between the groups, and Repeated Measures ANOVA to compare between different time points within the same group. Fisher’s Least Significant Difference (LSD) was used for post hoc analysis. The significance level was set at 0.01, and a post hoc power calculation was performed for the finally obtained sample size. With a total sample of 45 participants, divided into three equal groups, with four performed measurements, an effect size (f) of 0.5%, and an error probability (α) of 0.01, the achieved study power was 0.869. The IBM^®^ SPSS^®^ (version 20) statistical software (IBM, Armonk, NY, USA) was used for the analysis. The data analyst was blinded with regards to participants’ group allocation.

### 2.5. Ethical Considerations

The participants were informed about what to expect when using the custom-prepared mouthwashes during the designated study period. Participation was voluntary and the selected individuals were free to withdraw at any point of the study without affecting their health services provision at the institute. All personal or general information provided by the participants were confidential and to be used for research and educational purposes only. They were asked to sign an informed consent before the beginning of the study.

## 3. Results

The overall participants’ mean age was 20.7 ± 2 years. The mean scores for the PCR and GBI at baseline were 82.4 ± 19 and 85.8 ± 7, respectively, with no significant differences observed between the three mouthwash groups with regards to either parameter (*p* > 0.01) (Table 1). No harmful or side effects were reported by any of the participants throughout the study period.

**Figure 2:**
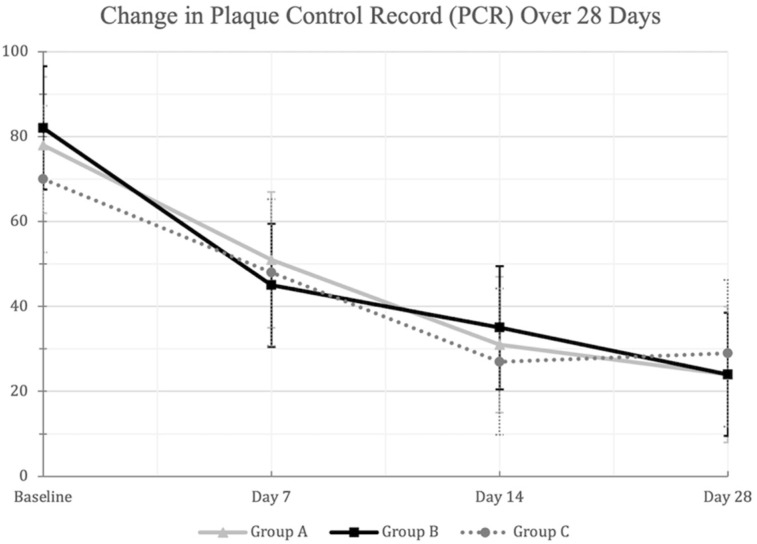
A line-chart showing the decrease in the mean (±SD) Plaque Control Record (PCR) scores in the three mouthwash groups at the different examination times. Changes from baseline to Day 28 were statistically significant within each mouthwash group at the 0.01 level using repeated measures ANOVA. [Group A: Green Tea (Warm MW), Group B: Green Tea (Hot-Ice MW), Group C: Placebo].

All mouthwash groups demonstrated significant reduction in PCR scores from Day 0 to Day 28 (*p* < 0.01) (Figure 2). However, no significant differences were observed between the mouthwash groups at any of the follow up examinations (*p* > 0.01) (Table 2).

**Figure 3:**
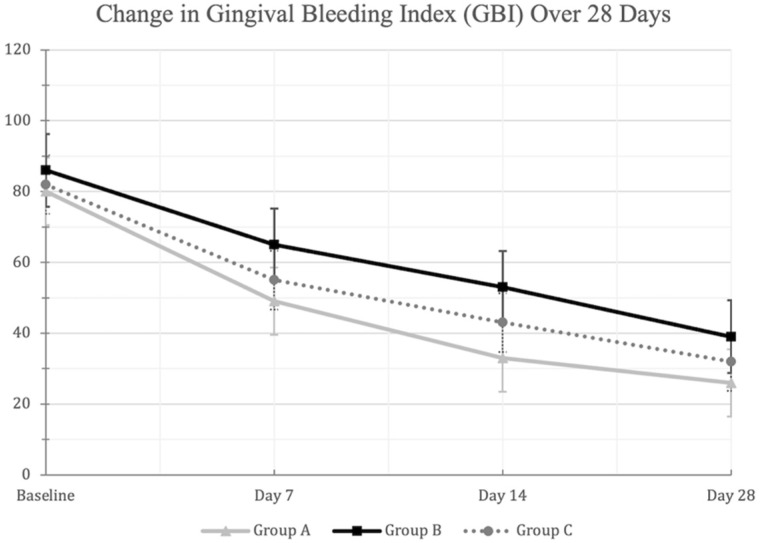
A line-chart showing the decrease in the mean (±SD) Gingival Bleeding Index (GBI) scores in the three mouthwash groups at the different examination times. Changes from baseline to Day 28 were statistically significant within each mouthwash group at the 0.01 level using repeated measures ANOVA. [Group A: Green Tea (Warm MW), Group B: Green Tea (Hot-Ice MW), Group C: Placebo].

Similarly, significant reduction in GBI scores were observed in all three mouthwash groups between days 0 and 28 (*p* < 0.01) (Figure 3). The warm green tea mouthwash group demonstrated significantly lower GBI scores on Day 14 compared to the other two groups (*p* < 0.01) (Table 2). Moreover, the warm green tea mouthwash group demonstrated significant reduction in GBI scores on days 7 and 28 when compared to the hot–ice mouthwash group (*p* < 0.01), but not to placebo (*p* > 0.01) (Table 3).

## 4. Discussion

The aim of this randomized controlled clinical trial was to compare the effect of green tea mouthwashes prepared at different temperatures on plaque-induced gingivitis when used as adjuncts to mechanical plaque control. Both test mouthwashes and placebo demonstrated significant reduction in dental plaque and gingival bleeding scores over the course of the study period. This was in line with what is mentioned in the literature and was expected since all participants received appropriate oral hygiene instructions and professional mechanical tooth cleaning within a short time period [2]. As part of the 11th European Workshop on Periodontology, on effective prevention of periodontal and peri-implant diseases, Chapple and co-workers concluded that professionally applied plaque control and reinforcement of oral hygiene leads to significant improvement in gingival inflammation and the lowering of plaque scores [8]. Moreover, the study by Venkateswara et al. also demonstrated that frequent rinsing positively contributes to the reduction of gingivitis. Nevertheless, the addition of green tea within mouthwash formulations was justified given its antioxidative, antimicrobial, antiviral, anticancer and anticariogenic properties [2].

A number of studies supported the effectiveness of green tea mouthwashes as antiplaque agents [12,25,26]. Hirasawa and co-workers [27] demonstrated in vitro that catechins of green tea, such as EGC, were found to inhibit the growth of certain periodontopathic bacteria including *Porphyromonas gingivalis*, *Prevotella intermedia*, and *Prevotella nigrescens*. Similarly, green tea catchains have expressed bactericidal effects against black-pigmented, Gram-negative anaerobic rods [27]. Findings from the current investigation are in line with such observations in reducing plaque scores over time. However, no differences between the studied mouthwash groups were observed. This could be due to the short duration of the study, as no comparison of the substantivity of green tea to chlorhexidine gluconate was performed [27]. Such findings raise questions regarding the cost-effectiveness and the prophylactic benefits of using green tea mouthwashes in the long-term, and motivates further research in this area. Moreover, future research may be directed towards comparison of the effectiveness of green tea mouthwashes and other recently trending approaches such as probiotic administration [28,29]. A systematic review reported that chlorhexidine and probiotic mouth rinses were equally effective in reducing plaque over 14 days [30]. Interestingly, probiotic mouth rinses were significantly more effective in reducing gingival inflammation than chlorhexidine [30].

Both tested green tea mouthwashes and the placebo contributed to the reduction of the gingival bleeding index over time. Interestingly, another study concluded that green tea-containing mouthwash is equally effective in reducing the gingival inflammation and plaque to chlorhexidine [12]. That study observed the effect of rinsing per se on gingivitis reduction. It may be concluded from that study and others that the added benefit of agents exhibiting anti-inflammatory properties may be in the short term when gingival inflammation is profound and requires comprehensive measures to be controlled.

A significant reduction in the gingival bleeding index was seen in the warm green tea mouthwash group compared to the hot green tea mouthwash group with added ice, consequently rejecting the posed statistical null hypothesis. This can be in part explained by the different impacts steeping conditions may have on green tea. A hot infusion of green tea has demonstrated rapid extractive power, but was associated with relevant compound degradation [20]. Cold infusion, on the other hand, extracted higher levels of healthy molecules with slow kinetics, antioxidant compounds, lower caffeine, less bitter taste and higher aroma, but required longer preparation time [20]. However, further prospective and experimental studies evaluating the changes in biological markers of inflammation and disease are required to reach more conclusive explanations.

### Limitations

This study only comprised female participants, which may have affected the generalizability of the findings. However, this decision was reached in an attempt to capture possible confounding factors such as hormonal imbalances, which may be encountered in daily clinical practice [31]. Furthermore, the strict inclusion and exclusion criteria, in addition to the random allocation of participants into the mouthwash groups may reduce the possible effect of confounders. Similarly, this trial exclusively recruited young adults, which was decided since younger aged individuals are known to develop gingival inflammation more rapidly compared to older individuals [32]. Another aspect was the relatively small sample size that may have limited the possibility to extrapolate the study conclusions into real life settings. This was approached by decreasing the significance level so as to avoid inflation of results or amplifying insignificant findings. The post hoc power calculation also revealed an acceptably achieved study power. In addition, the current study may well serve as an important preliminary investigation, from which further large-scale studies can be launched.

## 5. Conclusions

Within the study limitations, it can be concluded that green tea-made mouthwashes significantly reduced plaque and gingivitis when used as adjuncts to mechanical plaque control. The green tea mouthwash prepared in warm water demonstrated significantly higher efficacy in lowering gingival inflammation after two weeks compared to that prepared in hot water followed by ice addition. Further large-scale studies with longer follow-up comparing such home-made products with available gold standards are required to determine their actual value as adjuncts to periodontal therapy.

### Practical Significance

Findings of this study will help increase the awareness among health professionals and the community about the benefits of natural herbal products. Home-made remedies such as green tea mouthwashes may provide useful alternatives, especially for those with severe gingival inflammation and who cannot afford expensive chemical products to be used in conjunction with non-surgical periodontal therapy.

## Figures and Tables

**Table 1:** Medians, means and standard deviations of the age and the baseline Plaque Control Record and Gingival Bleeding Index in the total sample (N = 45) and the three mouthwash (MW) groups (n = 15).

Variable	All Participants (N = 45)		Group A: Green Tea (Warm MW) (n = 15)		Group B: Green Tea (Hot-Ice MW) (n = 15)		Group C: Placebo (n = 15)	
	Median	Mean ± SD	Median	Mean ± SD	Median	Mean ± SD	Median	Mean ± SD
Age	20	20 ± 2	20	20 ± 2	21	21 ± 2	20	20 ± 1
Plaque Control Record—PCR	80	77 ± 17	82	78 ± 17	86	82 ± 19	76	70 ± 13
Gingival Bleeding Index—GBI	82	82 ± 7	80	80 ± 7	85	86 ± 7	81	82 ± 6

**Table 2:** Comparison with regards to the Plaque Control Record (PCR) and Gingival Bleeding Index (GBI) between the three mouthwash (MW) groups at the different examinations. *p* values in bold fonts are statistically significant at the 0.01 level using One-Way ANOVA.

Variable	Group A: Green Tea (Warm MW) (n = 15)	Group B: Green Tea (Hot-Ice MW) (n = 15)	Group C: Placebo (n = 15)	*p*-Value
PCR (mean ± SD)				
Baseline Examination (Day 0)	78 ± 17	82 ± 19	70 ± 13	0.113
1st Examination (Day 7)	51 ± 22	45 ± 21	48 ± 23	0.767
2nd Examination (Day 14)	31 ± 9	35 ± 18	27 ± 7	0.236
3rd Examination (Day 28)	24 ± 10	24 ± 11	29 ± 21	0.633
GBI (mean ± SD)				
Baseline Examination (Day 0)	80 ± 7	86 ± 7	82 ± 6	0.053
1st Examination (Day 7)	49 ± 14	65 ± 10	55 ± 10	**0.001**
2nd Examination (Day 14)	33 ± 9	53 ± 8	43 ± 8	**<0.001**
3rd Examination (Day 28)	26 ± 11	39 ± 8	32 ± 14	0.013

**Table 3:** Post hoc analysis for the comparisons between the three mouthwash (MW) groups with regards to the Gingival Bleeding Index (GBI) at the different examinations. *p* values in **bold** fonts are statistically significant at the 0.01 level using Fisher’s Least Significant Difference (LSD).

Variable	Group A: Green Tea (Warm MW)	Group B: Green Tea (Hot-Ice MW)	Group C: Placebo
Baseline Examination (Day 0)			
Group A: Green Tea (Warm MW)	-	0.018	0.445
Group B: Green Tea (Hot-Ice MW)	0.018	-	0.099
Group C: Placebo	0.445	0.099	-
1st Examination (Day 7)			
Group A: Green Tea (Warm MW)	-	**<0.001**	0.125
Group B: Green Tea (Hot-Ice MW)	**<0.001**	-	0.025
Group C: Placebo	0.125	0.025	-
2nd Examination (Day 14)			
Group A: Green Tea (Warm MW)	-	**<0.001**	**0.004**
Group B: Green Tea (Hot-Ice MW)	**<0.001**	-	**0.001**
Group C: Placebo	**0.004**	**0.001**	-
3rd Examination (Day 28)			
Group A: Green Tea (Warm MW)	-	**0.003**	0.182
Group B: Green Tea (Hot-Ice MW)	**0.003**	-	0.088
Group C: Placebo	0.182	0.088	-

## Data Availability

The data presented in this study are available on request from the corresponding author. The data are not publicly available due to institutional privacy policies.
